# Deucravacitinib, an Oral, Selective, Allosteric Tyrosine Kinase 2 Inhibitor, in Asian Patients With Moderate to Severe Psoriasis: Improvements in Patient‐Reported Outcomes in a Randomized Trial

**DOI:** 10.1111/1346-8138.17834

**Published:** 2025-07-17

**Authors:** Jianzhong Zhang, Yangfeng Ding, Ping Wang, Linfeng Li, Weili Pan, Yan Lu, Hao Cheng, Xian Jiang, Ji‐Chen Ho, Shuping Guo, Seong Jun Seo, Linda Stein Gold, Andrew Blauvelt, Joe Zhuo, Yichen Zhong, Brandon Becker, Leona Liu, Subhashis Banerjee, Diamant Thaçi

**Affiliations:** ^1^ Department of Dermatology Peking University People's Hospital Beijing China; ^2^ Department of Dermatology Shanghai Skin Disease Hospital Shanghai China; ^3^ Department of Dermatology The First Affiliated Hospital of Chongqing Medical University Chongqing China; ^4^ Dermatology and Venereology Department Beijing Friendship Hospital Beijing China; ^5^ Department of Dermatology Zhejiang Provincial People's Hospital Zhejiang China; ^6^ Department of Dermatology Jiangsu Province Hospital Nanjing China; ^7^ Department of Dermatology and Venereology Sir Run Shaw Hospital, Zhejiang University School of Medicine Zhejiang China; ^8^ Department of Dermatology West China Hospital, Sichuan University Chengdu China; ^9^ Department of Dermatology Kaohsiung Chang Gung Memorial Hospital Kaohsiung Taiwan; ^10^ Department of Dermatology First Hospital of Shanxi Medical University Taiyuan China; ^11^ Department of Dermatology Chung‐Ang University Hospital Seoul South Korea; ^12^ Division of Dermatology Henry Ford Health System Detroit Michigan USA; ^13^ Oregon Medical Research Center Portland Oregon USA; ^14^ Bristol Myers Squibb Princeton New Jersey USA; ^15^ Institute and Comprehensive Center of Inflammation Medicine University of Lübeck Lübeck Germany

**Keywords:** clinical trial, patient‐reported outcome measures, quality of life, treatment outcome, tyrosine kinase inhibitors

## Abstract

POETYK PSO‐3, a 52‐week, double‐blind, phase 3 study, evaluated the efficacy and safety of deucravacitinib, an oral, selective, allosteric tyrosine kinase 2 inhibitor, in adult patients with moderate to severe plaque psoriasis in mainland China, Taiwan, and South Korea. Secondary and additional endpoints included improvement on two patient‐reported outcome measures: the Psoriasis Symptoms and Signs Diary (PSSD) total score and the Dermatology Life Quality Index (DLQI). Patients were randomized 1:2 to placebo or deucravacitinib 6 mg once daily; at week 16, patients receiving placebo crossed over to receive deucravacitinib. PSSD and DLQI score changes from baseline and response rates for achieving meaningful within‐patient change from baseline in PSSD total score (≥ 15 points) and DLQI of 0 or 1 (DLQI 0/1) were assessed over 52 weeks. In POETYK PSO‐3, 74 patients were randomized to placebo and 146 patients to deucravacitinib. At week 16, mean (95% confidence interval [CI]) PSSD total score changes from baseline were −1.9 (−6.9, 3.1) and −28.8 (−32.6, −25.0) in patients receiving placebo and deucravacitinib, respectively. At both weeks 16 and 52, the response rate for ≥ 15‐point meaningful change in PSSD total score (95% CI) was 73.3% (65.3, 80.3) in the group randomized to deucravacitinib. At week 16, mean (95% CI) DLQI changes from baseline were −1.7 (−3.1, −0.4) and −7.4 (−8.4, −6.4) in patients receiving placebo and deucravacitinib, respectively. In patients randomized to deucravacitinib, DLQI 0/1 response rates (95% CI) at weeks 16 and 52 were 36.4% (28.5, 44.4) and 44.7% (36.5, 52.9), respectively. Deucravacitinib was associated with meaningful and sustained improvements in psoriasis symptoms and signs and in quality of life in Asian patients with moderate to severe plaque psoriasis.

**Trial Registration:** ClinicalTrials.gov identifier: NCT04167462

## 1 Introduction

Psoriasis is a chronic, immune‐mediated inflammatory disease, and plaque psoriasis is the most common phenotype, accounting for more than 80% of cases [1]. Although a lower prevalence of disease has been observed in East Asian regions compared with Western European and North American groups (0.14% vs. 1.92% and 1.50%, respectively [2]), population differences in both disease presentation and treatment response have been described that render it crucial to examine treatments specifically in East Asian patients [3, 4]. Moreover, because psoriasis carries a high symptom burden that can profoundly impair patients' physical, social, and emotional quality of life (QoL) [5], it is important to consider patients' perspectives of treatment effects.

Deucravacitinib is an oral, selective, allosteric tyrosine kinase 2 inhibitor approved in the United States, European Union, South Korea, China, and other countries for the treatment of adults with moderate to severe plaque psoriasis [6, 7, 8, 9]. In the POETYK PSO‐3 trial, deucravacitinib was efficacious and well tolerated in adults with moderate to severe psoriasis from mainland China, Taiwan, and South Korea [10]. Additional endpoints included improvements in two patient‐reported outcome measures, Psoriasis Symptoms and Signs Diary (PSSD) and the QoL instrument, Dermatology Life Quality Index (DLQI). Here, efficacy with deucravacitinib on these two patient‐reported outcomes over 52 weeks in the POETYK PSO‐3 trial is reported.

## 2 Methods

### 2.1 Ethics

POETYK PSO‐3 was conducted in accordance with the principles of the Declaration of Helsinki and Good Clinical Practice, as defined by the International Council for Harmonization. The protocol was approved by the institutional review board or independent ethics committee at each study site, and all patients provided written informed consent before trial participation.

### 2.2 Trial Design and Participants

POETYK PSO‐3 was a 52‐week, randomized, double‐blind, phase 3 study conducted at 34 sites across mainland China, Taiwan, and South Korea. The full methodology has been published previously [10]. In summary, patients from these countries aged ≥ 18 years with stable moderate to severe plaque psoriasis (body surface area involvement ≥ 10%, Psoriasis Area and Severity Index ≥ 12, and static Physician Global Assessment score ≥ 3) were randomized 1:2 to receive placebo or deucravacitinib 6 mg once daily. Randomization was stratified by region (mainland China vs. Taiwan/South Korea) and by prior biologic therapy use for psoriasis or psoriatic arthritis (yes or no). At week 16, patients randomized to placebo crossed over to receive deucravacitinib, while patients randomized to deucravacitinib continued to receive the same treatment through week 52.

### 2.3 Outcome Measures

The PSSD is a validated instrument for assessing the severity of symptoms and patient‐reported signs in psoriasis [11]. Patients rate daily the severity of five skin symptoms and six skin signs associated with psoriasis over the past 24 h on an 11‐point numeric rating scale, where 0 = absent and 10 = worst imaginable. Symptom and sign summary scores range from 0 to 100; the total score represents the average of the summary scores. Weekly scores reflect the average of that week's daily scores. Three meaningful within‐patient change thresholds for the PSSD have been established: ≥ 15‐, ≥ 25‐, and ≥ 30‐point improvement from baseline in total scores [12]. For PSSD individual items, ≥ 2‐point score improvement from baseline reflects meaningful change [12].

The DLQI is a dermatology‐specific QoL instrument comprising 10 questions across six domains. Scored on a 4‐point Likert‐type scale, where 0 = not at all and 3 = very much, total scores range from 0 (no QoL impairment) to 30 (maximum QoL impairment) over the last week. The meaningful within‐patient change threshold for the DLQI has been determined to be at least a 4‐point improvement from baseline [13]. A DLQI of 0 or 1 (DLQI 0/1) indicates no effect on a patient's life. The DLQI was completed at baseline, weeks 1, 2, and 4, and every 4 weeks thereafter until week 52.

### 2.4 Assessments

#### 2.4.1 Continuous Outcomes

Adjusted mean changes from baseline for PSSD total score, PSSD individual item scores, and DLQI were derived using an analysis of covariance model with factors for region (mainland China vs. Taiwan or South Korea) and prior biologic use for psoriasis or psoriatic arthritis (yes or no), and with the baseline value included as a covariate. Modified baseline observation carried forward methods were used for missing data: the baseline observation was carried forward for patients who discontinued study treatment because of lack of response or adverse events, while patients who discontinued for other reasons had the last observation carried forward. Data are reported over 52 weeks for adjusted mean change from baseline in PSSD total score and DLQI, while adjusted mean score changes from baseline for PSSD individual items are reported at weeks 16 and 52.

#### 2.4.2 Binary Outcomes

Response rates from weeks 0 to 16 for achieving DLQI 0/1 in patients with baseline DLQI of ≥ 2 were compared using the Cochran–Mantel–Haenszel test. Data were stratified by region (mainland China vs. Taiwan or South Korea) and by prior biologic use for psoriasis or psoriatic arthritis (yes or no). The Clopper‐Pearson method was used to obtain 95% confidence intervals (CIs) for all other binary outcomes. Nonresponder imputation was used for missing data in all analyses of binary outcomes. Data are reported over 52 weeks for patients achieving meaningful change from baseline in PSSD total score and in DLQI, and for DLQI 0/1 response. Response rates for meaningful change from baseline on PSSD individual items are reported at weeks 16 and 52.

## 3 Results

The analysis included 74 patients randomized to placebo and 146 patients randomized to deucravacitinib (Figure S1). Across the entire study population, most patients were male (81.8%), and the mean (standard deviation [SD]) age at baseline was 40.6 (12.2) years. Demographics and baseline disease characteristics were similar across treatment groups (Table 1). The mean (SD) baseline PSSD total scores were 51.4 (23.5) and 55.2 (22.3) in patients randomized to placebo and deucravacitinib, respectively. Mean (SD) baseline DLQIs were 11.5 (7.8) and 12.8 (7.8) in patients randomized to placebo and deucravacitinib, respectively, indicative of a very large impact on patients' QoL. The PSSD bleeding item had the lowest baseline scores of the individual PSSD items across both treatment groups, with a mean (SD) score of 3.7 (2.9) in the placebo group and 2.9 (2.6) in the deucravacitinib group. Mean (SD) scores for the PSSD itch item were 6.1 (2.5) in the placebo group and 6.5 (2.4) in the deucravacitinib group; these were among the highest mean baseline scores for PSSD individual items.

**TABLE 1:** Demographics and baseline clinical characteristics.

Parameter	Placebo (*n* = 74)	Deucravacitinib (*n* = 146)
Age, mean (SD), years	41.2 (12.3)	40.3 (12.2)
Weight, mean (SD), kg	74.5 (14.4)	77.5 (15.8)
Male, *n* (%)	57 (77.0)	123 (84.2)
Disease duration, mean (SD) years	13.9 (9.0)	13.1 (8.5)
BSA involvement, mean (SD), %	33.4 (19.5)	34.1 (20.1)
PASI, mean (SD)	24.4 (10.9)	24.6 (10.4)
sPGA score, *n* (%)		
3	61 (82.4)	117 (80.1)
4	13 (17.6)	29 (19.9)
PSSD total score, mean (SD)	51.4 (23.5)	55.2 (22.3)
PSSD individual item score, mean (SD)		
Itch	6.1 (2.5)	6.5 (2.4)
Pain	4.1 (3.1)	4.8 (3.1)
Stinging	4.5 (3.1)	4.8 (2.9)
Burning	4.3 (3.1)	4.7 (2.9)
Tightness	5.9 (2.6)	5.9 (2.5)
Dryness	6.0 (2.6)	6.3 (2.3)
Cracking	5.1 (2.8)	5.4 (2.6)
Scaling	6.1 (2.4)	6.4 (2.2)
Shedding/flaking	6.1 (2.4)	6.3 (2.3)
Redness	5.6 (2.8)	6.2 (2.5)
Bleeding	2.9 (2.6)	3.7 (2.9)
DLQI, mean (SD)	11.5 (7.8)	12.8 (7.8)

Abbreviations: BSA, body surface area; DLQI, Dermatology Life Quality Index; PASI, Psoriasis Area and Severity Index; PSSD, Psoriasis Symptoms and Signs Diary; SD, standard deviation; sPGA, static Physician Global Assessment.

### 3.1 PSSD Total Score

**FIGURE 1:**
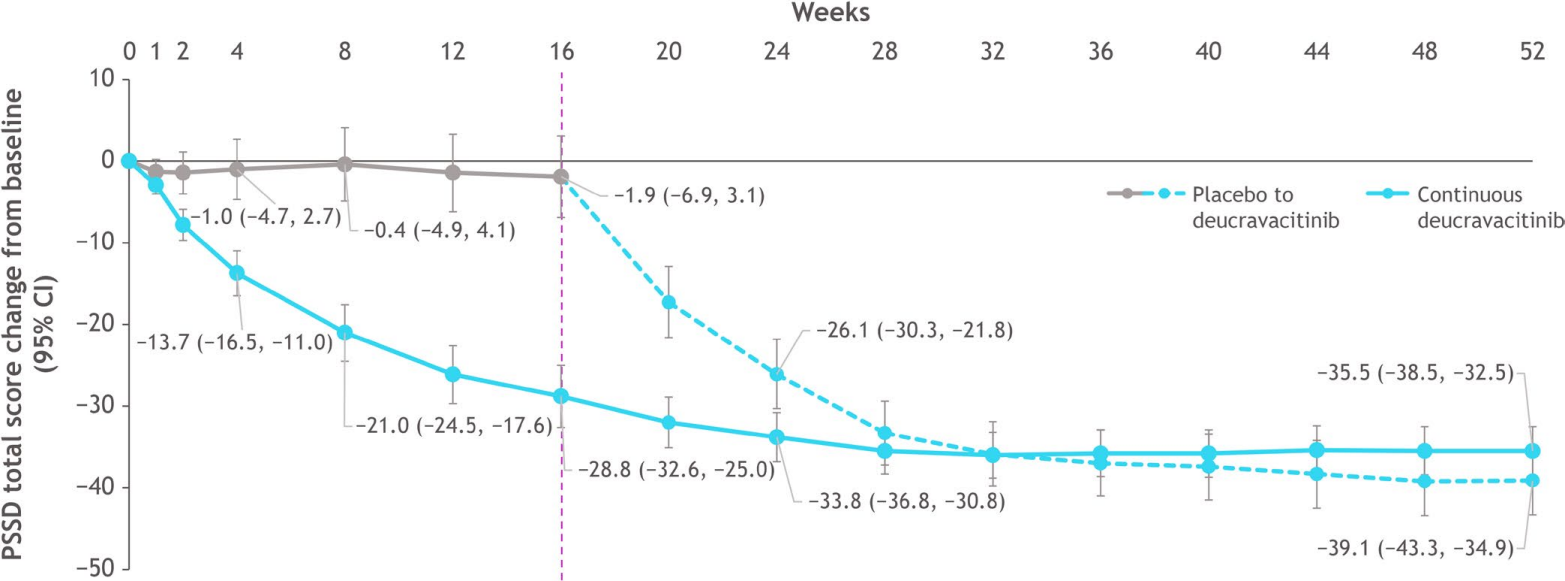
Change from baseline in PSSD total score over 52 weeks. At week 16, patients receiving placebo crossed over to receive deucravacitinib. CI, confidence interval; PSSD, Psoriasis Symptoms and Signs Diary.

Greater mean improvement from baseline in PSSD total score was observed as early as week 2 in the deucravacitinib versus the placebo group (*p* < 0.0001). At week 16, the adjusted mean (95% CI) score changes from baseline were −1.9 (−6.9, 3.1) in patients receiving placebo and −28.8 (−32.6, −25.0) in patients receiving deucravacitinib (Figure 1). Patients who received deucravacitinib continuously from baseline reported further improvements in PSSD total score from week 16 through week 32; these improvements were maintained through week 52. Patients who crossed over from placebo to deucravacitinib rapidly reported improvements in their psoriasis symptoms and signs after week 16. At week 52, the mean PSSD total score change from baseline (95% CI) was similar in each deucravacitinib treatment group (continuous deucravacitinib, −35.5 [−38.5, −32.5]; placebo to deucravacitinib, −39.1 [−43.3, −34.9]).

**FIGURE 2:**
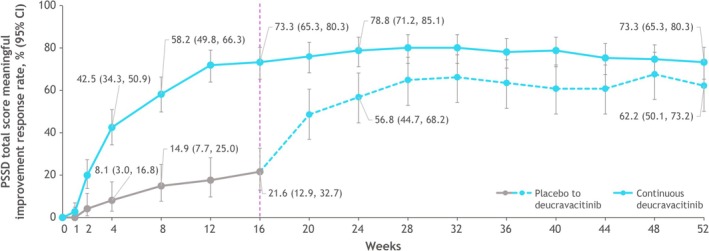
Response rates for ≥ 15‐point meaningful change from baseline in PSSD total score over 52 weeks. At week 16, patients receiving placebo crossed over to receive deucravacitinib. CI, confidence interval; PSSD, Psoriasis Symptoms and Signs Diary.

The response rate (95% CI) for ≥ 15‐points meaningful change in PSSD total score in patients treated with deucravacitinib was higher versus the placebo group by week 2 (19.9% [13.7, 27.3] vs. 4.1% [0.8, 11.4]). The difference in response rates (95% CI) between the treatment groups continued to increase until week 16 (21.6% [12.9, 32.7] vs. 73.3% [65.3, 80.3] in patients receiving placebo vs. deucravacitinib, respectively) (Figure 2). At week 52, the response rate (95% CI) was 73.3% (65.3, 80.3) in the group that had received deucravacitinib continuously from baseline, and 62.2% (50.1, 73.2) in the group that crossed over from placebo to deucravacitinib at week 16. Similar trends were observed at the ≥ 25‐ and ≥ 30‐point meaningful within‐patient change thresholds (Figure S2). At week 16, response rates were greater in patients randomized to deucravacitinib versus placebo, while week 52 response rates were similar between patients who received deucravacitinib continuously from baseline and those who crossed over to deucravacitinib treatment from placebo.

### 3.2 PSSD Individual Items

At week 16, adjusted mean change from baseline on each individual PSSD item was greater in the deucravacitinib versus placebo group (each *p* < 0.0001) (Figure S3A). Symptoms and signs continued to improve after week 16 in patients who received deucravacitinib continuously; at week 52, the greatest mean score improvements from baseline (95% CI) were observed on the itch (−4.2 [−4.5, −3.8]), scaling (−4.2 [−4.5, −3.9]), and shedding/flaking (−4.2 [−4.6, −3.9]) items (Figure S3B). In patients who crossed over from placebo to deucravacitinib, the greatest mean score improvements from baseline (95% CI) were observed on the itch (−4.4 [−4.8, −3.9]), dryness (−4.4 [−4.8, −3.9]), shedding/flaking (−4.5 [−5.0, −4.1]), and redness (−4.4 [−4.8, −3.9]) items (Figure S3B).

**FIGURE 3:**
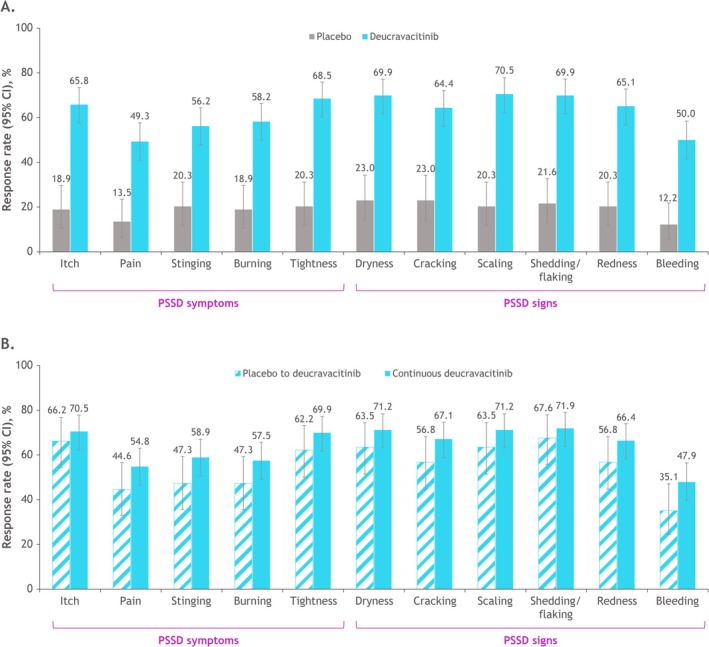
Response rates for meaningful change in scores on PSSD individual items (≥ 2‐point improvement from baseline) at week 16 (A) and week 52 (B). At week 16, patients receiving placebo crossed over to receive deucravacitinib. CI, confidence interval; PSSD, Psoriasis Symptoms and Signs Diary.

By week 4, the response rate (95% CI) for meaningful change on the PSSD itch item was higher in patients receiving deucravacitinib (33.6% [26.0 to 41.8]) versus placebo (5.4% [1.5, 13.3]). At week 16, the response rate (95% CI) was also higher in patients receiving deucravacitinib (65.8% [57.5, 73.4]) versus those receiving placebo (18.9% [10.7, 29.7]) (Figure 3A). Response rates for each individual PSSD item improved beyond week 16 in patients who received deucravacitinib continuously; at week 52, itch was the PSSD symptom item with the highest response rate (70.5% [62.4, 77.8]), while dryness and shedding/flaking were the PSSD sign items with the highest response rates (71.2% [63.2, 78.4] and 71.9% [63.9, 79.0], respectively) (Figure 3B). In patients who crossed over from placebo to deucravacitinib, week 52 response rates (95% CI) were highest in the itch (66.2% [54.3, 76.8]) and shedding/flaking (67.6% [55.7, 78.0]) items (Figure 3B).

### 3.3 DLQI

**FIGURE 4:**
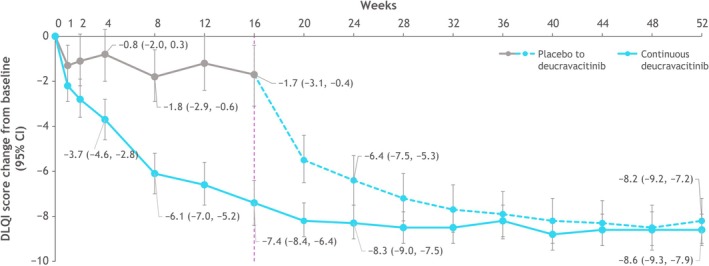
Change from baseline in DLQI over 52 weeks. At week 16, patients receiving placebo crossed over to receive deucravacitinib. CI, confidence interval; DLQI, Dermatology Life Quality Index.

Greater mean improvement from baseline in DLQI was observed as early as week 2 in the deucravacitinib group versus the placebo group (*p* = 0.009) (Figure 4). At week 16, the mean (95% CI) changes from baseline were −1.7 (−3.1, −0.4) in patients receiving placebo versus −7.4 (−8.4, −6.4) in patients receiving deucravacitinib. Score improvements in patients who received deucravacitinib continuously from baseline increased through week 28 and were maintained through week 52. Mean DLQI in patients who crossed over to deucravacitinib from placebo improved after week 16, with improvements reaching a plateau at week 44. At week 52, the improvements (95% CI) were comparable in each treatment group (continuous deucravacitinib, −8.6 [−9.3, −7.9]; placebo to deucravacitinib: −8.2 [−9.2, −7.2]).

**FIGURE 5:**
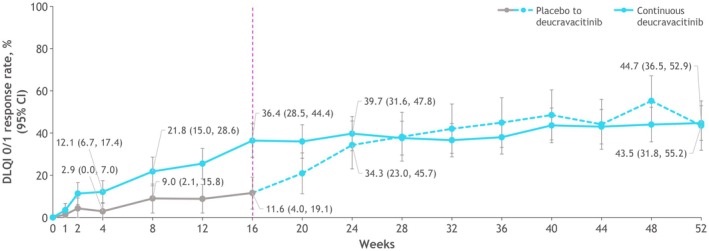
Response rates for DLQI 0/1 over 52 weeks. At week 16, patients receiving placebo crossed over to receive deucravacitinib. CI, confidence interval; DLQI 0/1, Dermatology Life Quality Index of 0 or 1.

Higher DLQI 0/1 response rates were seen with deucravacitinib versus placebo by week 4 (*p* = 0.03). The difference in response rates continued to increase through week 16 (Figure 5). The response rate in patients who received deucravacitinib continuously further improved from week 16 through week 40 and was maintained through week 52. At week 52, DLQI 0/1 response rates (95% CI) were similar between the continuous deucravacitinib treatment group (44.7% [36.5, 52.9]) and the placebo crossover to deucravacitinib group (43.5% [31.8, 55.2]).

By week 4, the response rates (95% CI) for ≥ 4‐point meaningful change in DLQI were higher in the deucravacitinib group (50.7% [42.3, 59.0]) versus the placebo group (28.4% [18.5, 40.1]) (Figure S4). The difference in response rates between the treatment groups increased until week 16. Patients who received deucravacitinib continuously from baseline maintained response until week 52, while the response rate in patients who crossed over from placebo to deucravacitinib increased after week 16, when all patients received deucravacitinib. At week 52, response rates (95% CI) in both treatment groups were similar (continuous deucravacitinib: 69.9% [61.7, 77.2]; placebo to deucravacitinib: 62.2% [50.1, 73.2]).

## 4 Discussion

In POETYK PSO‐3, treatment with deucravacitinib was associated with meaningful improvements in psoriasis symptoms and signs, as well as in QoL, in patients with plaque psoriasis from mainland China, Taiwan, and South Korea. These improvements were durable in patients who received deucravacitinib continuously from baseline and were reported at similar levels at week 52 in patients who crossed over to deucravacitinib from placebo at week 16. Of note, pruritus has been reported to be one of the most troubling symptoms for patients with psoriasis, with a particularly deleterious effect on QoL [14, 15, 16]. Approximately two‐thirds of patients reported meaningful improvement in PSSD itch item score at week 52.

These results are consistent with findings from the global POETYK PSO‐1 and PSO‐2 trials, respectively, where treatment with deucravacitinib was associated with significantly greater improvement from baseline in PSSD total scores (−28.8 vs. −27.8 and −30.1) and DLQI response (36.4% vs. 41.0% and 37.6%) at week 16 [17, 18, 19].

Though promising, this research is subject to some limitations. While the DLQI has been validated for use in Chinese patients with psoriasis and the PSSD has been validated in psoriasis [11, 20], the latter's psychometric properties have not been demonstrated in mainland China, Taiwan, or South Korea. The developers of the PSSD note, for example, that “some [US] patients may have difficulty distinguishing between pain, burning, and stinging,” [21] and it has not been determined whether these concepts remain robust and discrete in translation.

In conclusion, deucravacitinib was associated with meaningful improvements in patients' psoriasis symptoms and signs and in their QoL that were sustained through 52 weeks of treatment in patients from Asia.

## Conflicts of Interest

Jianzhong Zhang has served as a consultant for and received honoraria from Akeso Biopharma Co Ltd., Beijing Wenfeng Tianji Pharma Ltd., GSK (China) Investment Co Ltd., Keymed Biosciences (Chendu) Ltd., Kintor Pharmaceutical Ltd., Leo Pharma (Shanghai) Co Ltd., Lilly China, Novartis Pharmaceuticals (China), Pfizer Investment Co Ltd., Reistone Biopharma Co Ltd., Sanofi China, and Xian Janssen Pharmaceutical Co Ltd. Linda Stein Gold has served as a consultant, advisory board member, and/or speaker for AbbVie, Amgen, Arcutis, Aslan, Bausch Health, Boehringer Ingelheim, Bristol Myers Squibb, Dermavant, Dermira, Galderma, Incyte, Janssen, Leo Pharma, Lilly, Novartis, Pfizer, Regeneron, Sanofi Genzyme, Sun Pharma, and UCB. Andrew Blauvelt has served as a speaker (received honoraria) for Lilly and UCB; served as a scientific adviser (received honoraria) for AbbVie, Abcentra, Aclaris, Affibody, Aligos, Almirall, Alumis, Amgen, AnaptysBio, Apogee Therapeutics, Arcutis, Arena Pharmaceuticals, Aslan Pharmaceuticals, Athenex, Bluefin Biomedicine, Boehringer Ingelheim, Bristol Myers Squibb, Cara Therapeutics, Celldex Therapeutics, CTI BioPharma, Dermavant, EcoR1 Capital, Escient Pharmaceuticals, Evelo Biosciences, Evommune, Forte Biosciences, Galderma, Highlights Therapeutics, Incyte, Innovent, Janssen, Landos Biopharma, Leo Pharma, Lilly, Lipidio Pharma, Microbion Biosciences, Merck, Monte Rosa Therapeutics, Nektar, Novartis, Oruka, Overtone Therapeutics, Paragon Therapeutics, Pfizer, Q32 Bio, Rani Therapeutics, Rapt Therapeutics, Regeneron, Sanofi Genzyme, Spherix, Sun Pharma, Takeda Pharmaceuticals, TLL Pharmaceutical, TrialSpark/Formation Bio, UCB, Union Therapeutics, Ventyx Biosciences, Vibliome Therapeutics, and Xencor; and has served as a clinical study investigator (funds to institution) for AbbVie, Acelyrin, Allakos, Almirall, Alumis, Amgen, Arcutis, Athenex, Boehringer Ingelheim, Bristol Myers Squibb, Concert Pharmaceuticals/Sun Pharma, Dermavant, DermBiont, Evelo Biosciences, Evommune, Galderma, Incyte, Janssen, Leo Pharma, Lilly, Merck, Novartis, Pfizer, Regeneron, Sanofi, Sun Pharma, Takeda Pharmaceuticals, UCB, and Ventyx Biosciences. Joe Zhuo, Yichen Zhong, and Leona Liu are employees of and shareholders in Bristol Myers Squibb. Subhashis Banerjee and Brandon Becker were employees of and shareholders in Bristol Myers Squibb during the time of the study. Diamant Thaçi has received research support from and served as a principal investigator for AbbVie, Almirall, Amgen, Boehringer Ingelheim, Galderma, Janssen‐Cilag, Leo Pharma, Lilly, Merck Sharp & Dohme, Novartis, Pfizer, Regeneron, Sanofi, and UCB; has served as a consultant for AbbVie, Almirall, Galderma, La Roche Posay, Leo Pharma, New Bridge Pharmaceuticals, Novartis, Pfizer, Target RWE, and UCB; has served as a speaker for AbbVie, Almirall, Amgen, Janssen, Leo Pharma, Lilly, New Bridge Pharmaceuticals, Novartis, Pfizer, La Roche Posay, Sandoz‐Hexal, Sanofi, UCB, and Vichy Laboratories. The other authors declare no conflicts of interest.

## Supporting information


Data S1.

**Figure S1.** POETYK PSO‐3 patient disposition. Reproduced from Zhang et al. [10]. The Author(s) 2024. Published by Oxford University Press on behalf of British Association of Dermatologists. Creative Commons CC BY License https://creativecommons.org/licenses/by/4.0/. LTE, long‐term extension.

**Figure S2.** Response rates over 52 weeks for meaningful change from baseline in PSSD total score at thresholds of ≥ 25 points (A) and ≥ 30 points (B). At week 16, patients receiving placebo crossed over to receive deucravacitinib. CI, confidence interval; PSSD, Psoriasis Symptoms and Signs Diary.

**Figure S3.** Change from baseline in individual PSSD item scores at week 16 (A) and week 52 (B). At week 16, patients receiving placebo crossed over to receive deucravacitinib. CI, confidence interval; PSSD, Psoriasis Symptoms and Signs Diary.

**Figure S4.** Response rates for ≥ 4‐point meaningful change from baseline in DLQI over 52 weeks. At week 16, patients receiving placebo crossed over to receive deucravacitinib. CI, confidence interval; DLQI, Dermatology Life Quality Index.

## Data Availability

The Bristol Myers Squibb policy on data sharing may be found at https://www.bms.com/researchers‐and‐partners/independent‐research/data‐sharing‐request‐process.html.

## References

[1] A. W. Armstrong and C. Read , “Pathophysiology, Clinical Presentation, and Treatment of Psoriasis: A Review,” JAMA 323 (2020): 1945–1960. 32427307 10.1001/jama.2020.4006

[2] R. Parisi , I. Y. K. Iskandar , E. Kontopantelis , M. Augustin , C. E. M. Griffiths , and D. M. Ashcroft , “National, Regional, and Worldwide Epidemiology of Psoriasis: Systematic Analysis and Modelling Study,” British Medical Journal 369 (2020): m1590. 32467098 10.1136/bmj.m1590 PMC7254147

[3] J. Kim , C. H. Oh , J. Jeon , et al., “Molecular Phenotyping Small (Asian) Versus Large (Western) Plaque Psoriasis Shows Common Activation of IL‐17 Pathway Genes but Different Regulatory Gene Sets,” Journal of Investigative Dermatology 136 (2016): 161–172. 26763436 10.1038/JID.2015.378 PMC4731034

[4] J. E. Ferguson , E. W. Seger , J. White , and A. McMichael , “Racial/Ethnic Differences in Treatment Efficacy and Safety for Moderate‐To‐Severe Plaque Psoriasis: A Systematic Review,” Archives of Dermatological Research 315 (2023): 41–50. 35050396 10.1007/s00403-022-02324-4

[5] A. Armstrong , B. Bohannan , S. Mburu , et al., “Impact of Psoriatic Disease on Quality of Life: Interim Results of a Global Survey,” Dermatology and Therapy 12 (2022): 1055–1064. 35286611 10.1007/s13555-022-00695-0 PMC8918421

[6] Sotyktu [Package Insert] (Bristol Myers Squibb, 2022).

[7] Sotyktu [European Summary of Product Characteristics] (Bristol Myers Squibb EEIG, 2023).

[8] Sotyktu [Package Insert] (BMS Korea Pharmaceuticals Co., 2023).

[9] Sotyktu [Package Insert] (Bristol Myers Squibb (China) Investment Co., Ltd., 2023).

[10] J. Zhang , Y. Ding , P. Wang , et al., “Deucravacitinib, an Oral, Selective, Allosteric Tyrosine Kinase 2 Inhibitor, in Patients From China Mainland, Taiwan, and South Korea With Moderate to Severe Plaque Psoriasis: A Phase 3 Randomized Clinical Trial,” British Journal of Dermatology 192 (2025): 402–409. 39437312 10.1093/bjd/ljae406

[11] A. Armstrong , L. Puig , R. Langley , et al., “Validation of Psychometric Properties and Development of Response Criteria for the Psoriasis Symptoms and Signs Diary (PSSD): Results From a Phase 3 Clinical Trial,” Journal of Dermatological Treatment 30 (2019): 27–34. 28797188 10.1080/09546634.2017.1364694

[12] K. Papp , K. Gordon , B. Strober , et al., “Meaningful Change Thresholds for the Psoriasis Symptoms and Signs Diary: A Secondary Analysis of a Randomized Clinical Trial,” JAMA Dermatology 160 (2024): 204–209. 38117487 10.1001/jamadermatol.2023.5058 PMC10733845

[13] M. K. Basra , M. S. Salek , L. Camilleri , R. Sturkey , and A. Y. Finlay , “Determining the Minimal Clinically Important Difference and Responsiveness of the Dermatology Life Quality Index (DLQI): Further Data,” Dermatology 230 (2015): 27–33. 25613671 10.1159/000365390

[14] K. Jaworecka , M. Rzepko , L. Marek‐Józefowicz , et al., “The Impact of Pruritus on the Quality of Life and Sleep Disturbances in Patients Suffering From Different Clinical Variants of Psoriasis,” Journal of Clinical Medicine 11 (2022): 5553. 36233422 10.3390/jcm11195553 PMC9572740

[15] D. Globe , M. S. Bayliss , and D. J. Harrison , “The Impact of Itch Symptoms in Psoriasis: Results From Physician Interviews and Patient Focus Groups,” Health and Quality of Life Outcomes 7 (2009): 62. 19580674 10.1186/1477-7525-7-62 PMC2717072

[16] A. Reich , E. Hrehorów , and J. C. Szepietowski , “Pruritus Is an Important Factor Negatively Influencing the Well‐Being of Psoriatic Patients,” Acta Dermato‐Venereologica 90 (2010): 257–263. 20526542 10.2340/00015555-0851

[17] A. W. Armstrong , M. Gooderham , R. B. Warren , et al., “Deucravacitinib Versus Placebo and Apremilast in Moderate to Severe Plaque Psoriasis: Efficacy and Safety Results From the 52‐Week, Randomized, Double‐Blinded, Placebo‐Controlled Phase 3 POETYK PSO‐1 Trial,” Journal of the American Academy of Dermatology 88 (2023): 29–39. 35820547 10.1016/j.jaad.2022.07.002

[18] B. Strober , D. Thaçi , H. Sofen , et al., “Deucravacitinib Versus Placebo and Apremilast in Moderate to Severe Plaque Psoriasis: Efficacy and Safety Results From the 52‐Week, Randomized, Double‐Blinded, Program fOr Evaluation of TYK2 Inhibitor Psoriasis Second Phase 3 Trial,” Journal of the American Academy of Dermatology 88 (2023): 40–51. 36115523 10.1016/j.jaad.2022.08.061

[19] A. W. Armstrong , M. Augustin , J. L. Beaumont , et al., “Deucravacitinib Improves Patient‐Reported Outcomes in Patients With Moderate to Severe Psoriasis: Results From the Phase 3 Randomized POETYK PSO‐1 and PSO‐2 Trials,” Dermatology and Therapy 14 (2024): 2235–2248. 39080153 10.1007/s13555-024-01224-x PMC11333388

[20] Z. He , C. Lu , M. K. Basra , A. Ou , Y. Yan , and L. Li , “Psychometric Properties of the Chinese Version of Dermatology Life Quality Index (DLQI) in 851 Chinese Patients With Psoriasis,” Journal of the European Academy of Dermatology and Venereology 27 (2013): 109–115. 22145712 10.1111/j.1468-3083.2011.04371.x

[21] S. R. Feldman , S. D. Mathias , B. Schenkel , et al., “Development of a Patient‐Reported Outcome Questionnaire for Use in Adults With Moderate‐To‐Severe Plaque Psoriasis: The Psoriasis Symptoms and Signs Diary,” Journal of Dermatology & Dermatologic Surgery 20 (2016): 19–26.

