# Supplementary material for: Deucravacitinib, an Oral, Selective, Allosteric Tyrosine Kinase 2 Inhibitor, in Asian Patients With Moderate to Severe Psoriasis: Improvements in Patient‐Reported Outcomes in a Randomized Trial
Source: J Dermatol. 2025 Jul 17;52(9):1360–7. doi: 10.1111/1346-8138.17834 (PMC12411803; doi:10.1111/1346-8138.17834)

# Supporting Information

**Deucravacitinib, an oral, selective, allosteric tyrosine kinase 2 inhibitor, in Asian patients with moderate to severe psoriasis: improvements in patient-reported outcomes in a randomized trial**

Jianzhong Zhang, Yangfeng Ding, Ping Wang, Linfeng Li, Weili Pan, Yan Lu, Hao Cheng, Xian Jiang, Ji-Chen Ho, Shuping Guo, Seong Jun Seo, Linda Stein Gold, Andrew Blauvelt, Joe Zhuo, Yichen Zhong, Brandon Becker, Leona Liu, Subhashis Banerjee, Diamant Thaçi

**Figure S1.** POETYK PSO-3 patient disposition. Reproduced from Zhang J, et al. *British Journal of Dermatology*. 2024;ljae406. <https://doi.org/10.1093/bjd/ljae406>. © The Author(s) 2024. Published by Oxford University Press on behalf of British Association of Dermatologists. Creative Commons CC BY License <https://creativecommons.org/licenses/by/4.0/>. LTE, long-term extension.

**
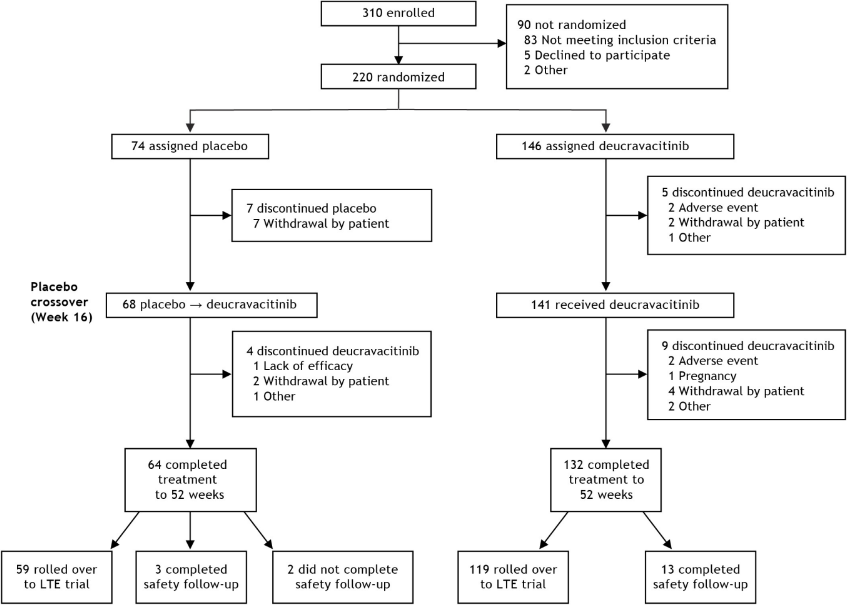
**

### **Figure S2.** Response rates over 52 weeks for meaningful change from baseline in PSSD total score at thresholds of ≥25 points (A) and ≥30 points (B). At week 16, patients receiving placebo crossed over to receive deucravacitinib. CI, confidence interval; PSSD, Psoriasis Symptoms and Signs Diary.

**
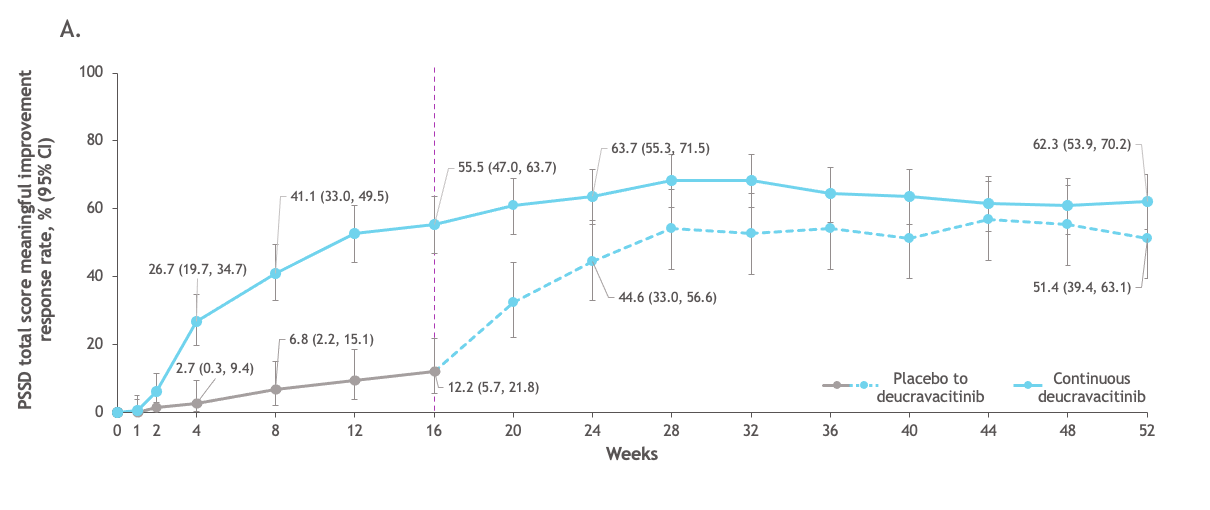
**

**
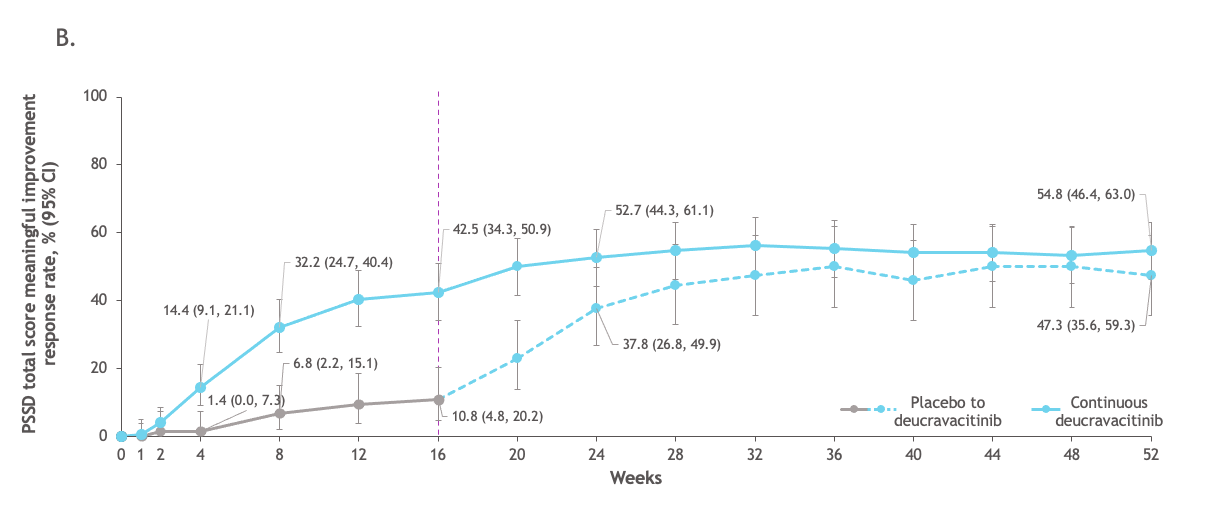
**

**Figure S3.** Change from baseline in individual PSSD item scores at week 16 (A) and week 52 (B). At week 16, patients receiving placebo crossed over to receive deucravacitinib. CI, confidence interval; PSSD, Psoriasis Symptoms and Signs Diary.


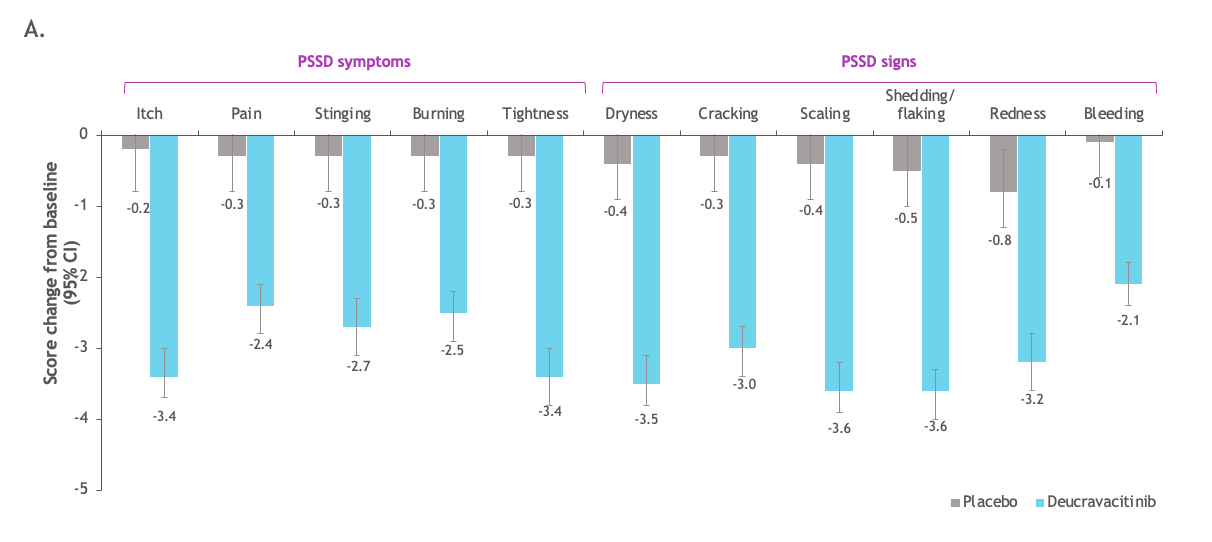


**
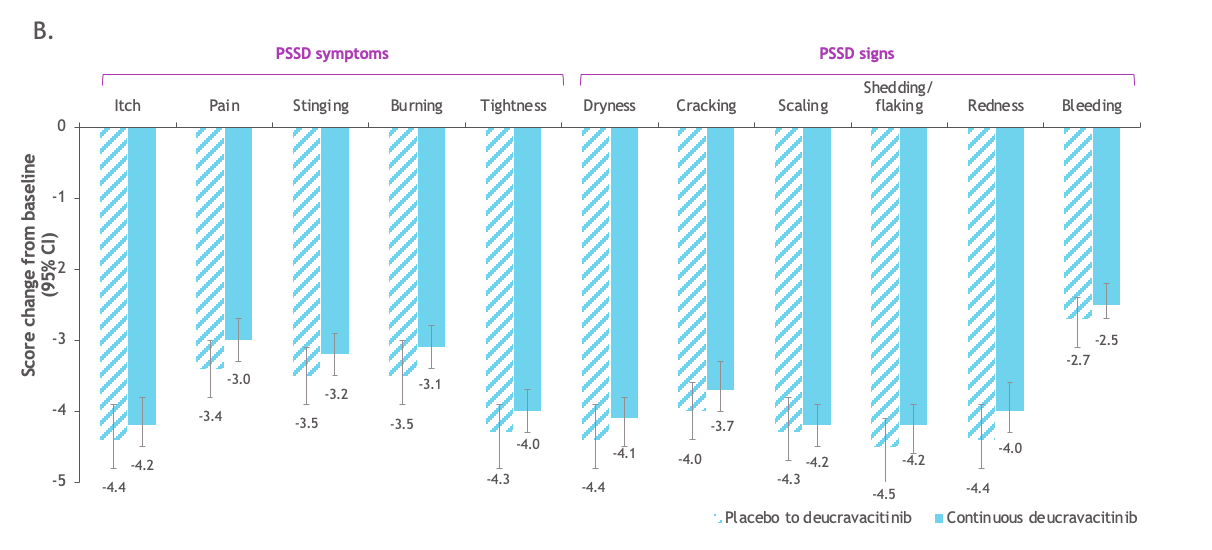
**

**Figure S4.** Response rates for ≥4-point meaningful change from baseline in DLQI over 52 weeks. At week 16, patients receiving placebo crossed over to receive deucravacitinib. CI, confidence interval; DLQI, Dermatology Life Quality Index.


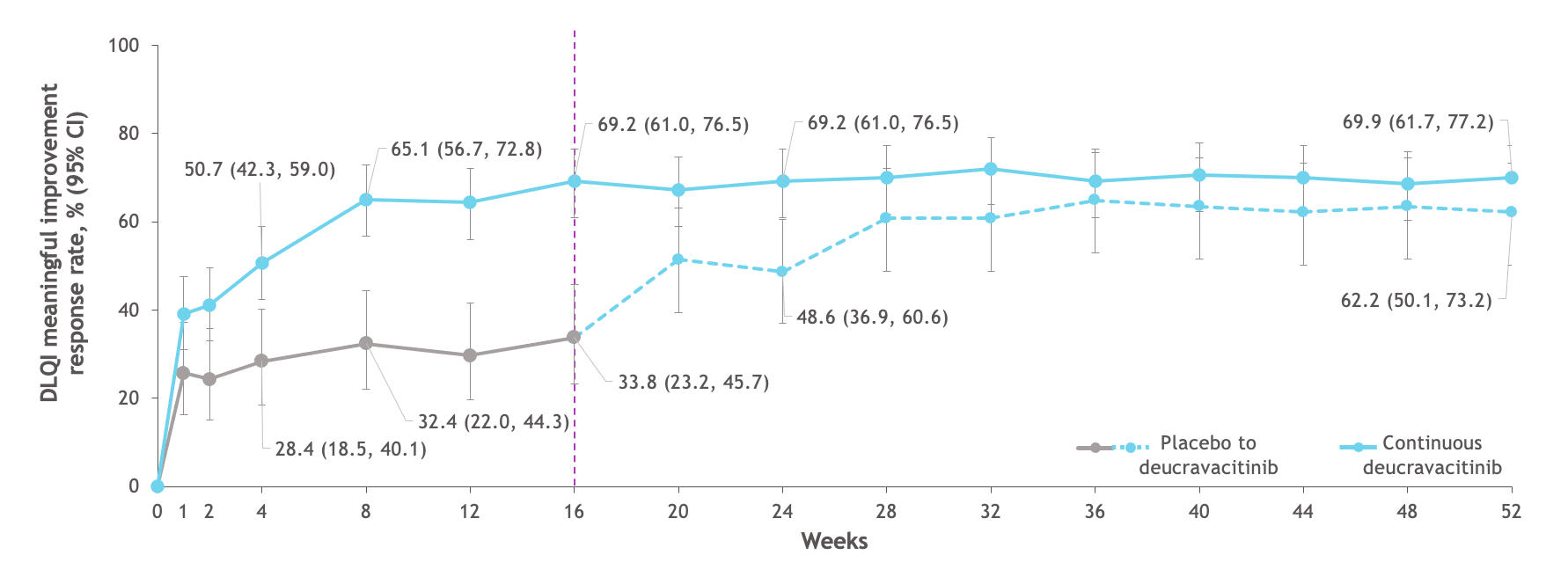

Supplement: Supplementary file 1 — Data S1. [file JDE-52-1360-s001.docx]
