# Supplementary figures and images for: Deucravacitinib, an Oral, Selective, Allosteric Tyrosine Kinase 2 Inhibitor, in Asian Patients With Moderate to Severe Psoriasis: Improvements in Patient‐Reported Outcomes in a Randomized Trial
Source: J Dermatol. 2025 Jul 17;52(9):1360–7. doi: 10.1111/1346-8138.17834 (PMC12411803; doi:10.1111/1346-8138.17834)

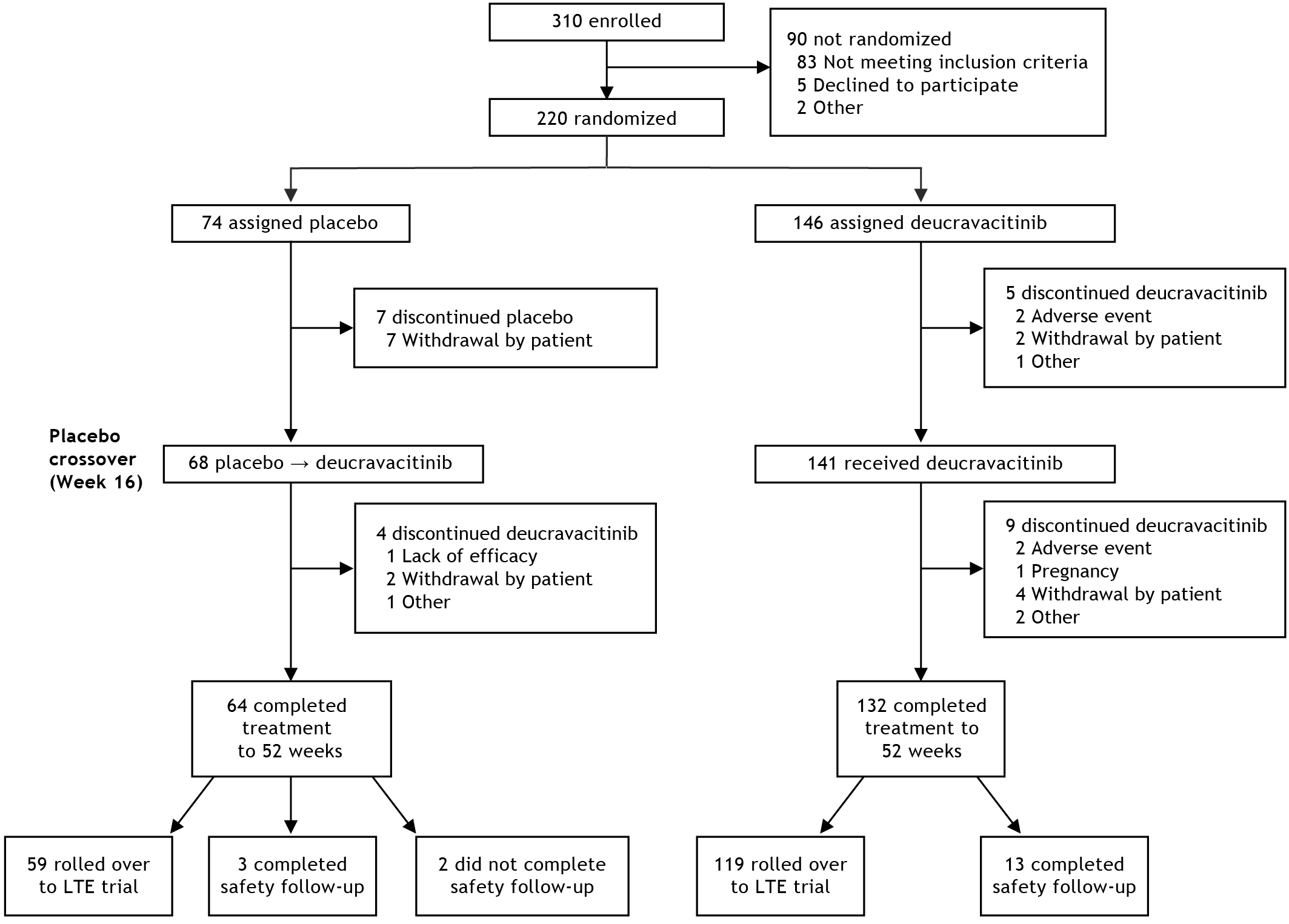

Supplement: Supplementary file 2 — Figure S1. POETYK PSO‐3 patient disposition. Reproduced from Zhang et al. [10]. The Author(s) 2024. Published by Oxford University Press on behalf of British Association of Dermatologists. Creative Commons CC BY License https://creativecommons.org/licenses/by/4.0/. LTE, long‐term extension. [file JDE-52-1360-s003.tif]

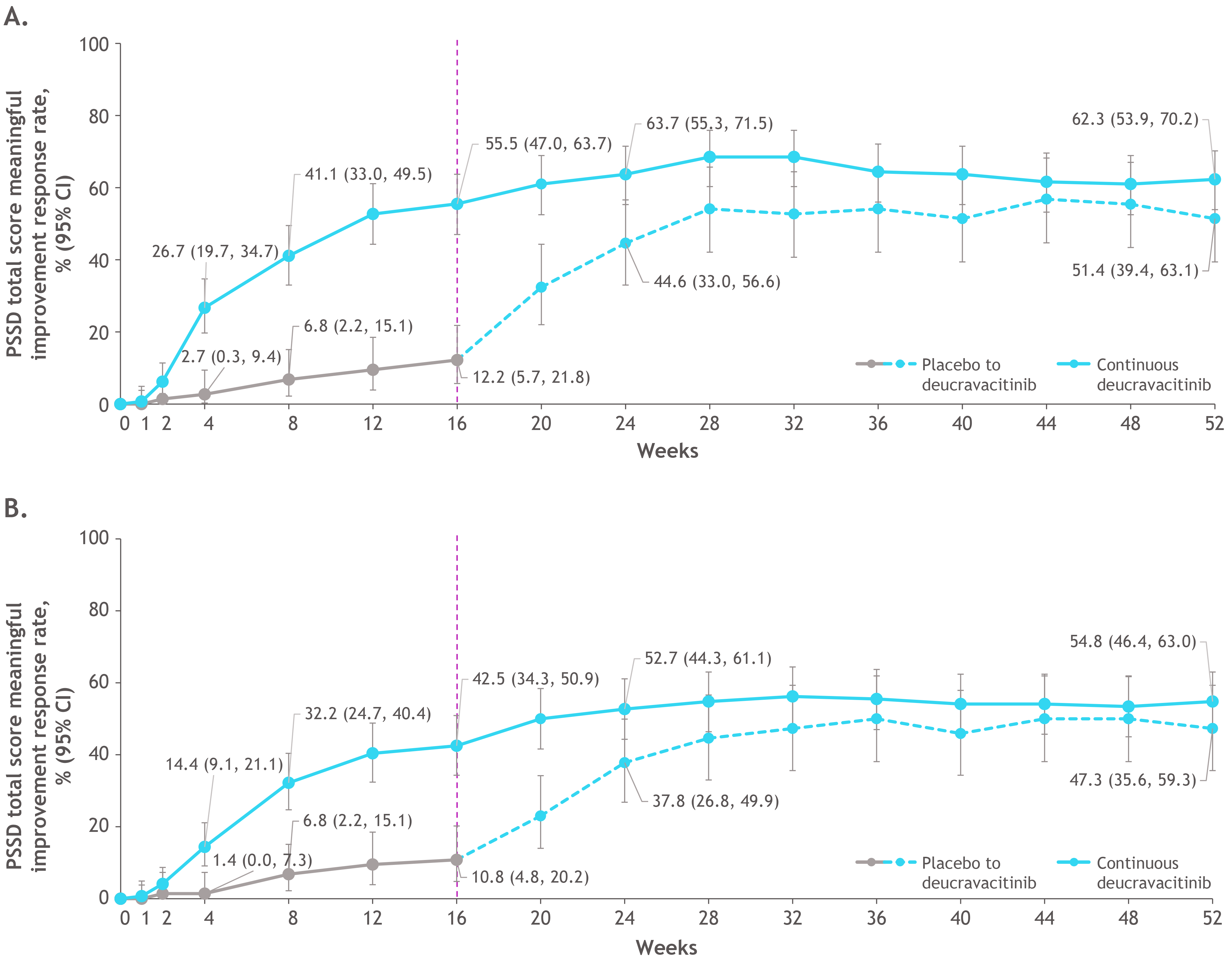

Supplement: Supplementary file 3 — Figure S2. Response rates over 52 weeks for meaningful change from baseline in PSSD total score at thresholds of ≥ 25 points (A) and ≥ 30 points (B). At week 16, patients receiving placebo crossed over to receive deucravacitinib. CI, confidence interval; PSSD, Psoriasis Symptoms and Signs Diary. [file JDE-52-1360-s002.tif]

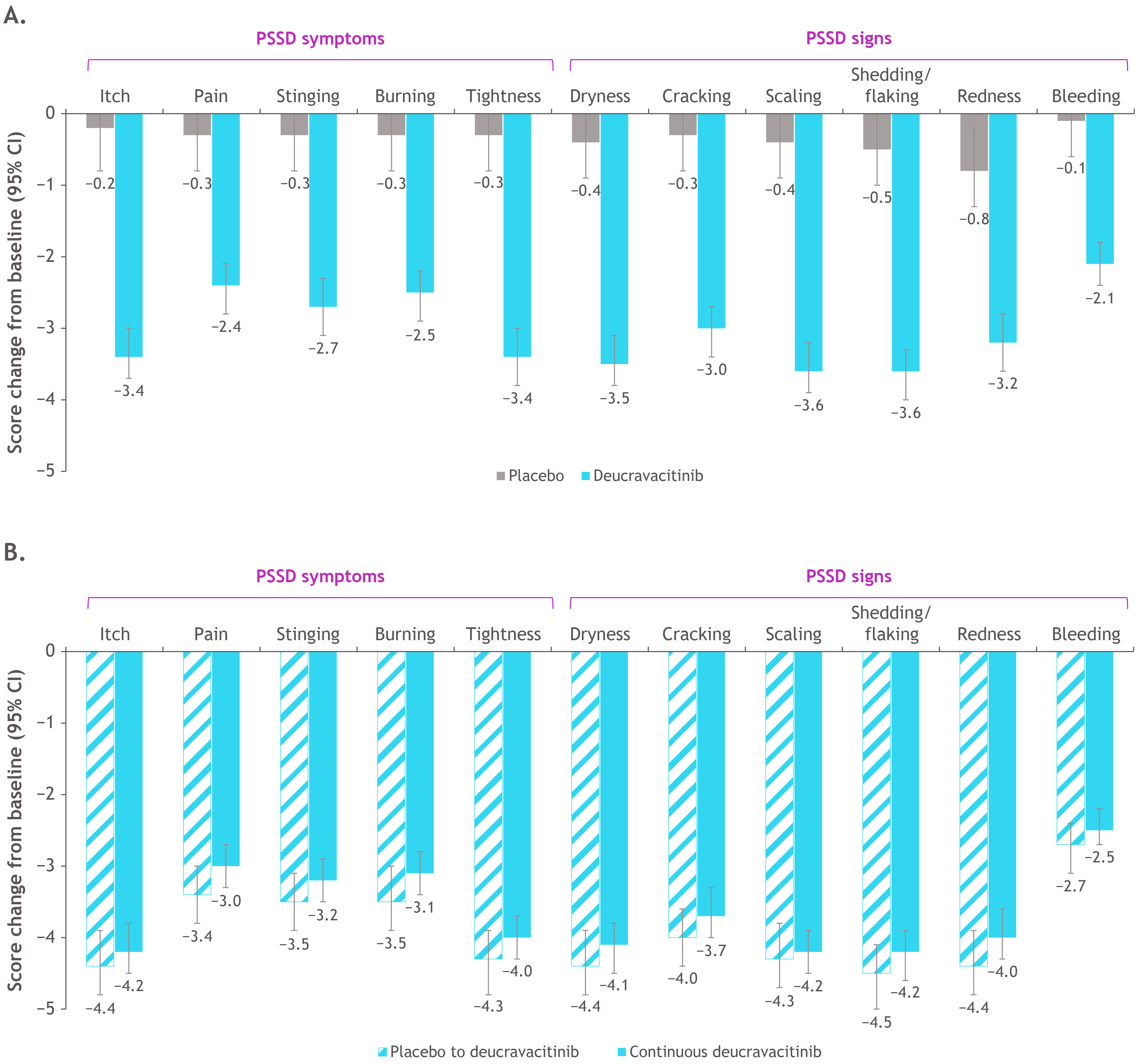

Supplement: Supplementary file 4 — Figure S3. Change from baseline in individual PSSD item scores at week 16 (A) and week 52 (B). At week 16, patients receiving placebo crossed over to receive deucravacitinib. CI, confidence interval; PSSD, Psoriasis Symptoms and Signs Diary. [file JDE-52-1360-s004.tif]

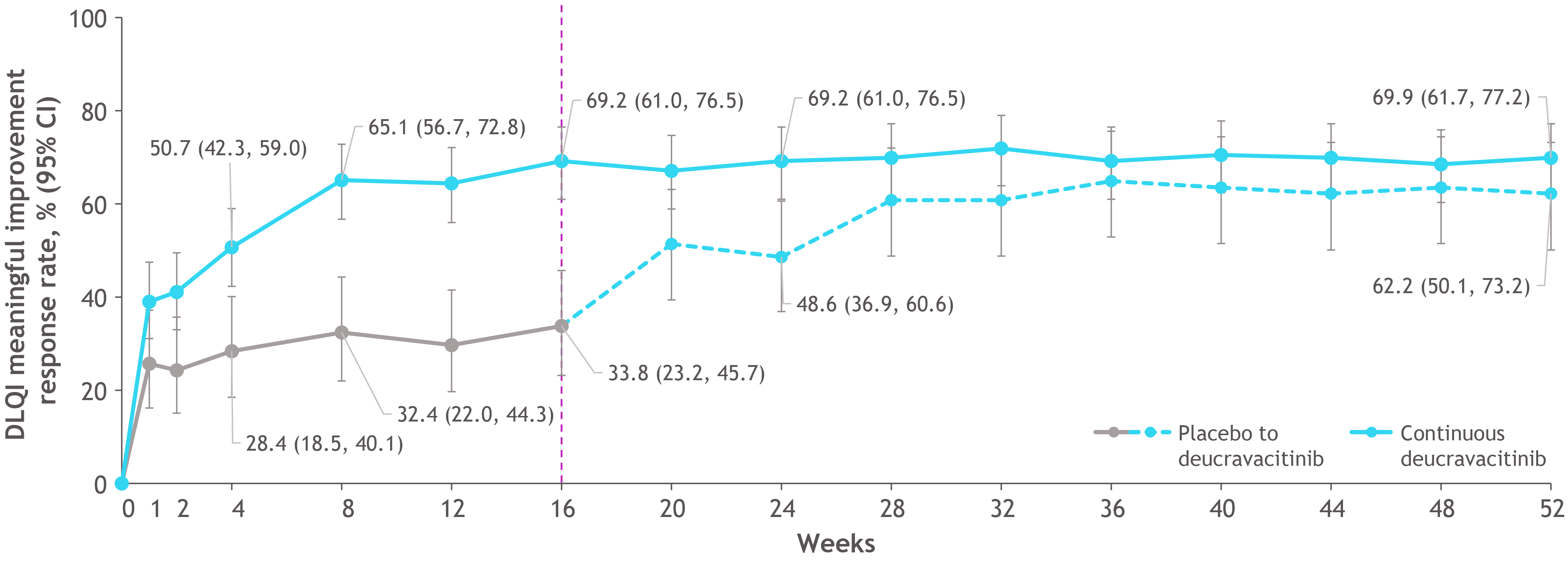

Supplement: Supplementary file 5 — Figure S4. Response rates for ≥ 4‐point meaningful change from baseline in DLQI over 52 weeks. At week 16, patients receiving placebo crossed over to receive deucravacitinib. CI, confidence interval; DLQI, Dermatology Life Quality Index. [file JDE-52-1360-s005.tif]
